# Neurocognitive, Clinical and Reelin Activity in Rehabilitation Using Neurofeedback Therapy in Patients with Schizophrenia

**DOI:** 10.3390/jcm13144035

**Published:** 2024-07-10

**Authors:** Renata Markiewicz, Agnieszka Markiewicz-Gospodarek, Mateusz Trubalski, Bartosz Łoza

**Affiliations:** 1 Occupational Therapy Laboratory, Medical University of Lublin, 7 Chodźki St., 20-093 Lublin, Poland; renatamarkiewicz@umlub.pl; 2 Department of Normal, Clinical and Imaging Anatomy, Medical University of Lublin, 4 Jaczewskiego St., 20-090 Lublin, Poland; 3 Student Scientific Association at the Department of Normal, Clinical and Imaging Anatomy, Medical University of Lublin, 20-090 Lublin, Poland; mateusztrub@gmail.com; 4 Department of Psychiatry, Medical University of Warsaw, 02-091 Warsaw, Poland; bartosz.loza.med@gmail.com

**Keywords:** reelin, schizophrenia, neurofeedback, rehabilitation, PANSS

## Abstract

**Introduction:** Reelin is a neuropeptide responsible for the migration and positioning of pyramidal neurons, interneurons, and Purkinje cells. In adulthood, it still supports neuroplasticity, especially dendritic spines formation and glutamatergic neurotransmission. Genetic studies have confirmed the involvement of reelin system failure in the etiopathogenesis of mental diseases, including schizophrenia. Given the role of reelin in brain cytoarchitectonics and the regularly observed reduction in its activity in prefrontal areas in cases of schizophrenia, dysfunction of the reelin pathway fits the neurodevelopmental hypothesis of schizophrenia, both as a biochemical predisposition and/or the ultimate trigger of psychosis and as a biosocial factor determining the clinical course, and finally, as a potential target for disease monitoring and treatment. **Aim:** The purpose of this study was to examine associations of the reelin blood level with clinical and neurocognitive parameters during an intensive, structured neurofeedback therapy of patients with schizophrenia. **Methods:** Thirty-seven male patients with paranoid schizophrenia were randomly divided into two groups: a group with 3-month neurofeedback as an add-on to ongoing antipsychotic treatment (NF, N18), and a control group with standard social support and antipsychotic treatment (CON, N19). The reelin serum concentration, clinical and neurocognitive tests were compared between the groups. **Results:** After 3-month trial (T2), the reelin serum level increased in the NF group vs. the CON group. The negative and general symptoms of PANSS (Positive and Negative Syndrome Scale) were reduced significantly more in the NF group at T2, and the d2 (d2 Sustained Attention Test) and BCIS (Beck Cognitive Insight Scale) scores improved only in the NF group. The AIS scores improved more dynamically in the NF group, but not enough to differentiate them from the CON group at T2. **Conclusions:** The clinical and neurocognitive improvement within the 3-month NF add-on therapy trial was associated with a significant increase of reelin serum level in schizophrenia patients.

## 1. Introduction

Schizophrenia is a complex disease whose treatment includes pharmacotherapy and rehabilitation interventions [1]. Among many rehabilitation interventions, an interesting form is neurofeedback, the therapeutic effect of which is focused on neurocognitive deficits, i.e., attention, memory, and concentration. To confirm the adopted thesis, this study verified the neurocognitive and clinical parameters and the changes in the reelin level under the influence of rehabilitation using neurofeedback therapy [2,3,4].

Neurofeedback therapy, employing real-time monitoring and self-regulation of neural activity, offers a promising strategy to address these neurocognitive issues [5,6,7]. By enabling individuals to actively modulate their brain activity, neurofeedback holds potential in mitigating attention deficits and enhancing working memory and executive functions. This patient-centric approach aligns with the goal of tailoring interventions to individual needs, fostering a more personalized and effective treatment realm. As we delve further into the impact of neurofeedback on specific neurocognitive aspects in schizophrenia, there lies the potential to significantly improve cognitive functioning and overall quality of life for those affected by this challenging mental health disorder [3,8]. Examining how neurofeedback therapy influences these broader clinical dimensions is crucial for evaluating its holistic effectiveness in symptom management [9,10]. Adding another element to this exploration is to consider the activity of the reelin pathway.

Reelin is a glycoprotein that plays an important role both during development (regulating migration and brain lamination) and in adulthood, maintaining synaptic functions. This glycoprotein plays several important roles in the development of the central nervous system (CNS), including mediating neuronal cell migration and proper brain lamination, while in the mature brain, it is involved in modulating synaptic functions [11]. The human reelin gene (RELN) is located on chromosome 7q22.1, and its protein product is an extracellular matrix protein [12].

Reelin expression is controlled by the transcription of its gene—RELN. This process is susceptible to regulation by various transcription factors that can activate or inhibit the transcription of the reelin gene in response to signals from the cellular environment, such as growth factors, cytokines, neurotransmitters, or other signaling proteins [13]. These external signals can influence the transcriptional activity of the reelin gene by activating intracellular signaling pathways. As a result, reelin expression is precisely regulated by several genetic and environmental factors, which allows it to respond to the changing developmental and functional needs of the brain [14]. Abnormal expression of reelin has been linked to various psychiatric disorders, highlighting the importance of this process for proper brain function.

Reelin, as a modulating biomarker of NMDA (N-methyl-D-aspartate), has been associated with the apolipoprotein E2 receptor (ApoER2) and the lipoprotein receptor (VLDLR), leading to a series of changes related to the phosphorylation and activation of impaired intracellular protein 1 (Dab-1). As a result of the interactions, a signaling cascade is activated, which includes, among others: PI3K kinase (phosphoinostide 3-kinase), Erk1/2 and GSK3 (glycogen synthase kinase-3), which are responsible for dendritic proliferation, triggering the process of dendritic growth and their so-called branching in the cerebral cortex [15,16,17,18,19]. 

Genetic studies have shown that there is a relationship between the level of reelin and the occurrence of classical mental diseases, like schizophrenia, bipolar disorder, and autism spectrum disorders [19,20,21,22,23]. Presumably, the occurrence of mental diseases is the result of reduced/suppressed expression of reelin in statu nascendi, which limits the proliferation of dendrites and impairs the cytoarchitectonics of neurogenesis. As a result of atopic dendrites, abnormal connections between neurons and abnormal neuronal migration occur [24,25,26,27,28]. The consequences of these disruptions result in neurocognitive deficits in patients with mental disorders. The question arises whether reelin dysregulation is a partially reversible process, and if so, whether the use of various methods improving neurocognitive processes (neurofeedback) can complement pharmacological treatment. This assumption was verified to a certain degree in this study.

Considered to be crucial in neurogenesis and synaptic plasticity [12,27,29,30,31], exploring the role of reelin during neurofeedback therapy provides a molecular perspective on the potential mechanisms occurring during symptom resolution in schizophrenia. Understanding how reelin activity is associated with symptom manifestation opens the way to uncovering the complex molecular dynamics contributing to the symptomatic aspects of this complex mental disorder [32,33].

The aim of this article was to analyze the level of reelin and neurocognitive parameters occurring under the influence of rehabilitation interventions using neurofeedback in patients diagnosed with schizophrenia.

## 2. Materials and Methods

### 2.1. Study Design

This study was a randomized, controlled 3-month trial reported with the use of the CONsolidated Standards of Reporting Trials (CONSORT) guidelines [34]. This trial is registered in the ISRCTN registry (Trial ID: ISRCTN78612833) where the full protocol can be found. 

Thirty-seven male patients with paranoid schizophrenia [35] were randomly divided into two groups: a group with intensive, structured neurofeedback, complementary to antipsychotic treatment (NF, N18), and a control group with standard social support and antipsychotic treatment (CON, N19). In this study, the minimal sample size (N) was calculated for the test power of the reelin serum variable in the range of not less than d = 0.8, which criterion is considered strong and adequate in behavioral sciences [36]. The N for 0.8 test power was set not less than 35.

### 2.2. Participants

The inclusion criteria (NF and CON groups) were patients’ consent; male gender; clinical diagnosis of paranoid schizophrenia [ICD]; age 18–50, right-handedness (writing); and no current neurological diseases, mental disability, or alcohol and/or psychoactive substance addiction. 

Members of both the NF and CON groups were recruited from among the participants at a city day-care center. They continued their previous antipsychotic treatment and usual clinical management. This study was limited only to male participants to reduce the risk of potential sex differences in neuropeptide levels that could not be corrected reliably between relatively small groups. Previous studies with a limited number of participants clearly indicated difficulties in interpreting the results in relation to sex [37,38]. Moreover, PANSS results can also be influenced by sex differences [39].

The subjects, after meeting the inclusion criteria, were randomly assigned to two groups (NF, CON), without the researchers participating in the assignment process. 

All recruited patients had remained relatively stable without experiencing active psychotic episodes for at least 18 months. These patients cannot be classified as clinically “residual” according to ICD-10-DCR criteria, as they were relatively young, active, and had experienced multiple episodes. They fit the pattern of episodic schizophrenia with stable or progressive development of negative symptoms between psychotic episodes (ICD-10-DCR: F20.01/F20.02) [40].

No current suicidal risk was diagnosed. 

As can be seen from Table 1, none of the main study parameters were statistically different at the baseline (T1). Almost all of the patients lived on a disability pension or other social benefits. A significant proportion of the participants smoked cigarettes: CON—68.42%, NF—66.67%.

During the experiments, all patients continued their former antipsychotic treatment (daily dose olanzapine equivalents in milligrams: NF vs. CON: M 16.44 SD 4.88 vs. M 18.47 SD 6.13). The antipsychotic treatment pattern was not changed during the experiment. All subjects were administered atypical antipsychotics (amisulpride, asenapine, aripiprazole, clozapine, olanzapine, quetiapine, or risperidone), and only some of them additionally received typical antipsychotics (flupenthixol, haloperidol, perazine, or zuclopenthixol). On average, half of the study participants were subjected to monotherapy (only with atypical antipsychotics): NF group—8/18, CON group—11/19. Polytherapy was delivered with either 2 or more atypical antipsychotics (NF 5/18, CON 5/19) or a combination of atypical and typical antipsychotics (NF 5/18, CON 3/19). A Chi-squared test for those three observations (atypical monotherapy, atypical polytherapy, and atypical/typical polytherapy) between NF and CON was insignificant (χ^2^ = 4.50, *p* = 0.999). None of the patients had taken anticholinergic drugs or participated in any long-term somatic treatment.

### 2.3. Outcome Measures

The examinations were performed twice, at the beginning (T1) and after a period of 3 months (T2).

#### 2.3.1. Neurocognitive Tests

##### d2 Sustained-Attention Test (d2)

The d2 test was administered to evaluate patients’ cognitive abilities, such as attention, sustained concentration, processing speed, and error correction skills [41]. This assessment involves 14 lines, each containing 47 characters. Participants have 20 s per line to mark all lowercase ‘d’s that have two apostrophes either above or below them. After each 20 s interval, the participant proceeds to the next line. The d2 test results yield various descriptive and complex indices [42,43]:

TN—the total number of letters marked both correctly and incorrectly; the speed of processing score;
E—raw score of omission and commission errors; the attention carelessness and confusion score;
E%—percentage of all errors; the overall accuracy score;
TN-E—the total number of items processed minus all errors; the impact of attention on the combined scores of speeds and accuracy as a perception ability;
CP—the concentration performance, the number of correctly processed items minus the commission errors;
FR—the fluctuation rate, which is based on the difference in the correct responses between the rows with the highest and lowest number of correct responses.

##### Beck Cognitive Insight Scale (BCIS)

The BCIS is an intricate 15-item self-report tool created to assess two dimensions of cognitive insight in individuals with psychosis: Self-Reflectiveness (9 items; BCIS-REF) and Self-Certainty (6 items; BCIS-CER) [44]. The composite Reflectiveness–Certainty Index (BCIS-INDEX) is calculated by subtracting the Self-Certainty score from the Self-Reflectiveness score, providing a balanced measure of cognitive insight [44].

##### Acceptance of Illness Scale (AIS)

The AIS comprises eight statements, each rated on a scale from 1 to 5 [45,46]. 

A higher score signifies better acceptance of the disease. The AIS evaluates not only the patient’s awareness of having schizophrenia but also their perception of the disease through its consequences.

##### General Self-Efficacy Scale (GSES)

The GSES aims to assess the adaptive potential to challenging environmental demands by taking corrective action [47,48]. The original, 10-item version was used in this study.

##### Color Trials Test (CTT)

The CTT consists of two distinct tasks [49]. In the first task (CTT-1), the respondent connects circles in a numerical sequence from 1 to 25. The second task (CTT-2) involves connecting numbers from 1 to 25 while alternating between pink and yellow circles, ignoring a distractor colour. CTT-1 and CTT-2 were designed to assess sustained and selective attention, visuospatial skills, and motor speed. Additionally, CTT-2 evaluates Stroop-like effects based on mental flexibility by constantly challenging the executive memory [50]. The Interference Index (CTT-II) is calculated as the difference between the times for CTT-2 and CTT-1, divided by the CTT-1 time, providing insight into the increased relative time required to complete a more cognitively demanding task.

#### 2.3.2. PANSS

Clinical parameters were assessed using the Positive and Negative Syndrome Scale (PANSS) [51]. This 30-item interview is an operational tool designed to offer a balanced representation of positive, negative, and general psychopathology in patients with schizophrenia. It includes three subscales and provides a total score that reflects the severity of psychopathology.

#### 2.3.3. Laboratory

The serum level of reelin peptide was determined immunoenzymatically with the ELISA technique [product of the WUHAN company, catalog number E2134H, name ELISA Kit FOR Reelin, unit 96TST, distributor BIOKOM-biologia cell, BIOKOM, Poland].

The reelin level was determined at 07:00 a.m. (pg/mL), using a non-contact method of blood sampling into a clot tube.

### 2.4. Neurofeedback Therapy

Neurofeedback (NF) training sessions were conducted twice a week over a three-month period. The training program was designed to gradually increase the task difficulty based on individual progress. The galvanic skin response (GSR) method was utilized, which includes two components: the general tonic-level electrodermal component (skin conductance level, SCL) and the phasic component (skin conductance responses, SCRs). These components are crucial diagnostic parameters for managing mental disorders. In GSR-NF, they can be used as references to modulate the patient’s emotional state according to current needs [51]. The GSR-NF training sessions were conducted in the CENTER (relaxation), BALANCE (concentration), and INSECTS (self-control) modules using a Digi-Track apparatus (EEGDigiTrack Biofeedback- EEG + SpO_2_ + HR). NF trainings were performed in accordance with the approved schedule; training was conducted in a sound-proof room always at a specified time, mainly after breakfast. Patients were requested not to drink coffee or smoke for one hour before the training. The measurements were made by the exosomatic method with DC (direct current) using electrodes placed on the index and ring fingers of the left hand and connected to the device displaying the successive training modules. The training exercises in the individual modules were displayed on a monitor, and patients followed the given instructions to perform them. In the CENTER exercise, participants had to guide bubbles appearing on the screen into a circle at the centre by achieving relaxation through controlled breathing and heart rate modulation. Greater relaxation enabled faster task completion and progression to the next module level. The BALANCE module aimed to enhance concentration, requiring participants to maintain maximum focus to place and balance a ball in the centre of a tilting board. The INSECT module focused on achieving internal balance between cognitive and executive functions. Participants needed to identify and click on moving and hidden insects on the screen, with the insects’ slow movement indicating gradual attainment of internal balance, facilitating task completion. The GSR apparatus recorded neurophysiological changes, assessing participants’ psychophysical condition based on skin resistance.

The training time was set by the computer program and was 5 min for the CENTER and BALANCE modules and 10 min for the INSECT module. Prior to NF training and after the 3-month program the level of clinical and neurocognitive deficits was assessed.

The NF program was enriched through structured rehabilitation activities aimed at enabling behavioral training and overexpression of neurocognitive competences acquired through NF sessions.

The NF training was supported with five main modules: social training, motivation/planning capacity, cognitive training, computer-assisted training (perception, attention, reasoning), and a creativity module. It emphasized not only teaching skills, but also improving metacognition and solving social problems. It was a largely balanced, psychosocial therapy programme, and the achievement of any skill was not an inclusion or exclusion criterion. Our rehabilitation program referred to some extent to the cognitive remediation therapy principles developed by Wykes et al., showing a predictive potential related to the patient’s ability to function in the community [52].

The program was administered to the NF group and was neither hierarchically nor sequentially organized. Its purpose was to alter daily routines through additional social activities, fostering team competencies, providing training in social roles, enhancing personal acceptance, and bolstering independence. Structured activities were conducted in daily blocks of up to 8 h, excluding weekends. The daily schedule included group activities such as assertiveness training and role-playing techniques, psychotherapy, psychoeducation, cognitive training, art therapy, physiotherapy, sports, social events, communal cooking, entertainment activities, and relaxation training. Every day, there was at least one session of group psychotherapy or psychoeducation.

The therapy schedule for the CON group consisted of typical counselling, unstructured visits, and a medication continuation schedule, as well as social worker supervision.

### 2.5. Statistical Analyses

The values of the investigated variables were presented as means and standard deviations. The sociological and demographic parameters were presented as numbers and percentages. The results were compared using conservative, non-parametric Mann-Whitney U and Chi-squared tests, as well as repeated measures analysis of variance (ANOVA/MANOVA). The Bonferroni method was conducted to correct for multiple comparisons. Differences were considered statistically significant at *p* < 0.05. Analyses were performed using Statistica 13.3.

### 2.6. Ethical Issues

The study protocol was approved by the local Bioethics Committee—approval no. KE-0254/35/2016. All of the patients invited to take part in the study gave their written informed consent.

## 3. Results

A comparison of the final effects of NF therapy at T2, i.e., over a period of 3 months, is presented in Table 2.

Table 2 presents the results of the 3-month trial. While none of the baseline parameters at T1 differentiated the NF and CON groups, at T2, differences occurred in many domains, i.e., in terms of serum reelin level (NF > CON increase), in almost all PANSS dimensions (NF > CON clinical improvement) with the exception of positive symptoms, and finally, patients from the NF group obtained more favourable results on two neurocognitive tests, i.e., d2 scores (three of its six subscales) and BCIS scores (three of its three subscales).

A repeated measures ANOVA was used to compare mean scores across multiple observations. A comparison of the main effects of NF vs. CON therapy over a period of 3 months is presented in Table 3.

Regarding the neurocognitive tests, two of them accumulated significant changes between the NF and CON groups. In the d2 test, these were three subscales, i.e., Errors, Errors% and CP, while in the BCIS test these were REF, CER, and the BCIS Index. The alpha levels of these scores were strong enough that internal analyses of the subscales of these tests remained significant (except for d2-Errors) using Bonferroni corrections (0.008 and 0.170, respectively). The GSES total score in the repeated-measures analysis was numerically significant, but this condition did not persist after applying the Bonferroni correction.

## 4. Discussion

Schizophrenia is a recurrent mental illness characterized by neurocognitive deficits, like impaired attention, working memory, and problem solving [53,54,55]; limitations in the ability to interpret and process social-emotional information [56]; and it seem to be related to structural and functional changes in the brain [4]. Since each episode of the disease causes a deepening of these deficits, and thus the deterioration of the general social functioning of patients, several studies have aimed at implementing interventions strengthening cognitive functions and making the treatment more comprehensive [57,58,59,60]. The search for new methods to enhance cognitive functions is important because pharmacotherapy does not meet expectations and its effect in terms of neurocognitive improvement is estimated at a low-moderate level [61]. Antipsychotics act mainly symptomatically, while neurocognitive functions are more embedded in the etiopathogenesis of schizophrenia [62]. Therefore, patients need therapeutic methods that have a deeper effect on the core process of “dysconnectivity” or “underconnectivity” and rehabilitation of the richness of human experiences and activities, not just suppressing symptoms such as delusions or hallucinations with antipsychotics [54,55,56,57,58,59,60,61,62,63]. Rehabilitation of patients with schizophrenia has been shown to stimulate reelin promoter activity and increase neurocognitive connectivity in the CNS [54].

Previous research confirmed the effectiveness of various interventions through biochemical analyses, specialized software, fMRI, and psychological tests. Most therapeutic trials are based on neuroplasticity, highlighting the brain’s ability to adapt and change based on environmental demands and experiences [60]. The evidence shows that learning reorganizes and expands neural networks. The effectiveness of interventions for cognitive functions in schizophrenia is mainly linked to the prefrontal area [64]. Some studies indicate that early interventions can improve cognitive functions and protect grey matter [54,65,66,67]. 

When analyzing the effects of interventions, reference should be made to the neuromodulation of CNS and the biomarkers involved in this process. One of them is reelin, with a special role in neuronal migration, as well as its stimulation of dendritogenesis and effective synaptic transmission [30,68,69]. Reelin’s involvement in signalling is mediated by two leading lipoprotein receptors: the apolipoprotein receptor (ApoER2) and the very low-density lipoprotein receptor (VLDLR). The effect of the interaction of these receptors with reelin is the activation of a signalling cascade, which also involves the Dab1 adapter [16,33]. Studies confirmed the relationship between inducing signalling and modulating transmission and cognitive processes based on functional coupling with the NMDA receptor [70,71,72,73]. The reelin information pathway is additionally modified by the action of various isoforms of this neuropeptide [33].

Possible disturbances in the functioning of the reelin cascade are diverse and multi-level and may lead to neurological, psychiatric, and neurodegenerative disorders [33,74,75,76,77,78]. Reelin activity in the brain in patients diagnosed with schizophrenia, bipolar disorder and depression may be reduced by 30–50% [79]. 

Therefore, it seems reasonable to take actions aimed at improving cognitive functions based on various behavioral trainings. One of the new concepts is cognitive remediation (CR) training, which is based on the principle of neuroplasticity effects. Ho et al. (2020) verified this main principle in clinical work [54]. Epigenetic, neurocircuitry and behavioral indicators were analyzed in patients diagnosed with schizophrenia (N = 18) undergoing CR and in the control group without CR (N = 17). The measurements included the analysis of the reelin and BDNF promoters in samples collected with oral swabs. The CR program included 60 h of computer-assisted neurocognitive training and 45 h of social–cognitive group sessions. Neurocognitive outcomes were assessed using a standard BACS battery. The level of CNS connectivity was analyzed with fMRI. The program confirmed the relationship of active rehabilitation (CR) with the activity of the reelin pathway, increase in functional connectivity in the frontal and frontotemporal areas, and improvement of neurocognitive functions. 

Similar results were obtained by Eack et al. (2012), who also analyzed the effects of CR cognitive enhancement therapy on frontotemporal brain connectivity in a randomized, controlled study involving people in the early stage of schizophrenia [67]. The two-year study period covered a group of 41 patients diagnosed with schizophrenia (in remission) and their fMRI data, including functional connectivity, were analyzed annually. CR training increased connectivity between the frontal and temporal areas of the brain and improved neurocognitive functions, especially problem solving and emotion processing [80].

In another study, Marimoto et al. (2018) assessed the neuroanatomical effects in patients diagnosed with schizophrenia undergoing CR training (twice-weekly computer-assisted sessions and weekly group meetings for a period of 12 weeks). The comparative analysis was performed twice, before inclusion in the study and after a period of 12 weeks, based on the MRI morphometric analysis. That study confirmed that cognitive remediation therapy induced neurocognitive improvement through hippocampal plasticity. An increased right hippocampal volume was positively associated with verbal fluency scores [81].

The results of the present comparison are consistent with the articles presented above in terms of the basic observation that intensive rehabilitation, partly based on cognitive training and partly on social skills training, can effectively stimulate biochemical and anatomical processes leading to the improvement of higher cognitive functions [54,80,81]. In general, the neurofeedback therapy used in our present study is like CR therapy. We are most consistent with the trial of Ho et al. (2020) [54], where, similar to us, the hypothesis of the influence of reelin on neuroplasticity and cognitive functioning was positively verified. What differs between the cited works and our analysis is the selection of patients in terms of the phase of schizophrenia deterioration/symptom dynamics. Although in compared studies [54,80,81] no baseline clinical differences were found between the study and control groups (similarly to ours), detailed analyses of clinical symptoms were not presented on a multi-week basis, apparently considering that the research endpoints were limited to neurocognitive effects. Meanwhile, the primary target of our analysis was the schizophrenic symptomatology—these turned out to be significantly and specifically different between the NF and CON groups and was associated with an increase in the serum level of reelin during neurofeedback therapy. The conclusions presented in these publications prove that the brain is a complex communication network that does not function in isolation. This mutual communication between individual structures, referred to by neurobiologists as brain connectivity, is of great importance in the comprehensive rehabilitation of people diagnosed with schizophrenia [82,83,84,85].

Undoubtedly, further genetic and biochemical studies are necessary to determine the exact role of reelin and other markers involved in the signalling pathway. It seems important to verify the obtained data in a larger group of participants, which will confirm our results.

## 5. Conclusions

1. An intensive, structured neurofeedback therapy as an add-on to ongoing antipsychotic treatment was related to an increase in the reelin serum level (NF group) in contrast to control patients (CON group).
2. This study indicates a possible, comprehensive relationship between the effects of neurofeedback add-on therapy with an increase in reelin serum level and an improvement of neurocognitive functions as well as a reduction in negative and general symptoms of PANSS in patients with schizophrenia.

## 6. Study Limitations

1. The number of respondents is a starting point for further, extended research.
2. Measurements of reelin levels performed repeatedly during rehabilitation interventions can undoubtedly facilitate the analysis of the reelin level profile and determine its modification and expression. The analysis of the reelin profile may constitute an interesting interpretation from the point of view of neurophysiology.
3. The group of subjects (NF/CON) was limited only to men, and this was an intentional assumption to eliminate intersexual hormonal differences and thus differences in possible reactions. Interesting research can be conducted by comparing groups of women with the analyzed group of men.
4. When qualifying many patients for the study, additional blinding may be introduced in the NF group under study; in the case of a small group of patients, the introduction of such a procedure could limit the achievement of significant results.
5. Demonstrating the positive impact of NF as a method increasing the effect of traditional rehabilitation can be confirmed by other neurotrophic markers such as: BDNF or neuropeptide S. From the point of view of neurophysiology, a comparison of the obtained level of reelin with other markers could confirm various interesting research hypotheses.
6. Clearly, further genetic and biochemical studies are needed to determine the specific role of various receptors in the signaling pathway. Perhaps they will confirm preliminary findings that the reelin pathway influences the formation or stabilization of anatomical synapses.

## 7. Research Implications

1. Neurorehabilitation as an integral element of comprehensive psychiatric treatment and should be implemented during the first episode of the disease.
2. Rehabilitation interventions carried out by medical workers can improve the level of cognitive and social functioning of sick people.
3. Mental health care workers should implement rehabilitation interventions at every level of treatment based on modern neurorehabilitation tools, including biofeedback.

## 8. Strengths of the Study

1. Directing future clinical research toward a multi-aspect approach to neurogenesis.
2. Emphasizing the importance of rehabilitation in the comprehensive treatment of schizophrenia.
3. Searching for new neurorehabilitation methods (biofeedback) that can be used in people with mental disorders to improve their cognitive and social functioning.

Nonetheless, the results of this study should be interpreted with caution, as no confounding variables were included in the analysis.

## Figures and Tables

**Table 1:** Initial (T1) parameters and pairwise comparisons (Mann–Whitney test) for NF and CON groups.

Variable	NF		CON		NF vs. CON	
	M	SD	M	SD	U	*p*
d2-TN	309.89	80.65	335.16	50.25	143.00	0.403
d2-E	77.50	47.99	94.79	44.84	132.50	0.248
d2-E%	24.87	14.94	29.06	14.48	138.00	0.323
d2-TN-E	232.39	71.98	240.37	70.27	165.50	0.879
d2-CP	102.11	42.86	125.47	28.58	121.00	0.133
d2-FR	13.61	6.03	15.74	9.01	151.50	0.564
BCIS-REF	22.33	4.72	22.95	5.30	162.50	0.808
BCIS-CER	14.33	2.52	16.47	3.82	117.50	0.107
BCIS-INDEX	8.00	4.04	6.47	4.86	140.50	0.362
AIS-Total	23.33	7.85	27.16	8.15	125.00	0.172
CTT-1	61.67	32.24	56.11	26.10	157.50	0.693
CTT-2	124.11	54.14	117.37	55.28	155.50	0.649
CTT-II	1.16	0.75	1.13	0.61	167.50	0.927
GSES-Total	28.78	4.80	32.21	6.39	113.00	0.081
PANSS Positive	19.11	3.51	19.90	4.64	162.00	0.796
PANSS Negative	14.56	3.26	15.16	3.70	154.00	0.616
PANSS General	25.39	3.71	27.53	10.37	164.50	0.855
PANSS Total	49.50	7.79	52.63	15.05	155.00	0.638
Age of first hospitalization (years)	26.06	28.90	4.21	6.82	126.00	0.176
Hospitalizations	5.78	3.67	4.95	2.68	157.00	0.682
Education (ISCED grades)	2.67	1.03	2.90	0.99	151.00	0.554
Antipsychotics in milligrams (equivalents of olanzapine)	16.44	4.88	18.47	6.13	134.50	0.274
Reelin (pg/mL)	4.40	2.24	3.75	1.62	144.00	0.421
BMI (kg/m^2^)	28.78	3.28	27.28	3.21	118.00	0.111
Age (years)	36.61	6.45	37.84	10.36	162.50	0.808

NF—neurofeedback group, CON—control group, d2-TN—total answers, d2-E—errors, d2-E%—percentage of all errors, d2-TN-E—total number minus all errors, d2-CP—concentration performance, d2-FR—fluctuation rate, BCIS—Beck Cognitive Insight Scale (Reflectiveness, Certainty, Index), AIS—Acceptance of Illness Scale, CTT—Color Trails Test (trail 1, trail 2, interference index), GSES—General Self-Efficacy Scale, PANSS—Positive and Negative Syndrome Scale (Positive, Negative, General, Total), ISCED—International Standard Classification of Education, BMI—body mass index, M—mean, SD—standard deviation, U—Mann-Whitney U-test, *p*—*p*-value significance at *p* < 0.05.

**Table 2:** Neurocognitive, reelin and PANSS results (T2) of neurofeedback 3-month therapy (NF) versus standard therapy (CON).

Test	Subtest	CON		NF		U	*p*
		M	SD	M	SD		
d2	TN	364.79	70.40	320.28	79.18	119.50	0.121
	E	102.90	29.76	63.44	33.10	68.50	**0.002**
	E%	29.45	11.35	20.22	10.32	94.00	**0.020**
	TN-E	261.90	75.18	256.83	75.49	155.50	0.649
	CP	149.47	78.74	103.33	45.45	101.50	**0.036**
	FR	15.63	10.29	12.28	2.80	160.00	0.750
CTT	CTT-1	53.46	0.88	54.17	28.98	162.50	0.808
	CTT-2	104.77	44.72	113.44	42.23	140.50	0.362
	CTT-II	1.10	0.56	1.37	0.82	147.50	0.485
BCIS	BCIS-REF	22.37	5.89	25.72	3.14	101.00	**0.035**
	BCIS-CER	17.90	4.05	13.50	2.31	62.50	**0.001**
	BCIS-INDEX	4.47	6.05	12.22	3.17	46.00	**0.000**
AIS	Total	28.37	7.85	28.56	7.15	164.50	0.855
GSES	Total	32.42	6.29	29.89	4.52	121.50	0.137
Reelin (pg/mL)		3.08	1.44	5.74	2.64	64.00	**0.001**
PANSS	Total	56.68	16.41	42.67	8,47	65.50	**0.001**
	Positive	9.58	2.73	8.33	1.82	121.50	0.137
	Negative	17.47	4.53	12.11	3.66	61.50	**0.001**
	General	29.63	10.82	22.22	3.78	65.50	**0.001**

d2—d2 Sustained Attention Test, TN—total number of letters marked, E—errors, E%—percentage of all errors, TN-E—total number of items processed minus errors, CP—concentration performance, FR –fluctuation rate, CTT—Color Trails Test, CTT-II—Interference Index, BCIS—Beck Cognitive Insight Scale, BCIS-REF—self-reflectiveness subscale, BCIS-CER—self-certainty subscale, AIS—Acceptance of Illness Scale, GSES—General Self-Efficacy Scale, PANSS—Positive and Negative Syndrome Scale, U—Mann-Whitney U-test, *p*-value bolded at *p* < 0.05. Statistical significance is indicated in bold.

**Table 3:** Analysis of variance (ANOVA/MANOVA) in repeated-measures design: Treatment groups (NF vs. CON) × time (T1 vs. T2).

		Wilks’ Test	F	df	Error	*p*
Reelin	pg/mL	0.636	3.046	6	72	**0.010**
PANSS	Positive	0.667	2.800	6	72	**0.017**
	Negative	0.254	11.790	6	72	**0.000**
	General	0.326	9.020	6	72	**0.000**
	Total	0.242	12.375	6	72	**0.000**
CTT	CTT-1	0.941	0.368	6	72	0.897
	CTT-2	0.940	0.382	6	72	0.889
	CTT-II	0.939	0.385	6	72	0.887
d2	TN	0.726	2.088	6	72	0.065
	E	0.630	3.119	6	72	**0.009**
	E%	0.585	3.695	6	72	**0.003**
	TN-E	0.859	0.951	6	72	0.465
	CP	0.572	3.865	6	72	**0.002**
	FR	0.915	0.546	6	72	0.772
BCIS	REF	0.609	3.378	6	72	**0.005**
	CER	0.581	3.749	6	72	**0.003**
	INDEX	0.548	4.210	6	72	**0.001**
AIS	Total	0.765	1.721	6	72	0.128
GSES	Total	0.693	2.419	6	72	**0.035**

F—F Statistics, df—degrees of freedom, Error—variability within groups/unexplained error, d2—d2 Sustained Attention Test, TN—total number of letters marked, E—errors, E%—percentage of all errors, TN-E—total number of items processed minus errors, CP—concentration performance, FR—fluctuation rate, CTT—Color Trails Test, CTT-II—Interference Index, BCIS—Beck Cognitive Insight Scale, BCIS-REF—self-reflectiveness subscale, BCIS-CER—self-certainty subscale, AIS—Acceptance of Illness Scale, GSES—General Self-Efficacy Scale, PANSS—Positive and Negative Syndrome Scale. *p*-value bolded at *p* < 0.05. Statistical significance is indicated in bold.

## Data Availability

The data that support the findings of this study are available from the corresponding author upon reasonable request.
